# The genome sequence of a braconid wasp, *Aleiodes testaceus *(Telenga, 1941)

**DOI:** 10.12688/wellcomeopenres.22738.1

**Published:** 2024-07-26

**Authors:** Gavin R. Broad, Iona Cunningham-Eurich, Natural History Museum Genome Acquisition Lab, Darwin Tree of Life Barcoding collective, Wellcome Sanger Institute Tree of Life Management, Samples and Laboratory team, Wellcome Sanger Institute Scientific Operations: Sequencing Operations, Wellcome Sanger Institute Tree of Life Core Informatics team, Tree of Life Core Informatics collective, Darwin Tree of Life Consortium

**Affiliations:** 1 Natural History Museum, London, England, UK; 2 University College London, London, England, UK

**Keywords:** Aleiodes testaceus, braconid wasp, genome sequence, chromosomal, Hymenoptera

## Abstract

We present a genome assembly from an individual female *Aleiodes testaceus* (braconid wasp; Arthropoda; Insecta; Hymenoptera; Braconidae). The genome sequence spans 110.70 megabases. Most of the assembly is scaffolded into 19 chromosomal pseudomolecules. The mitochondrial genome has also been assembled and is 28.0 kilobases in length. Gene annotation of this assembly on Ensembl identified 10,520 protein-coding genes.

## Species taxonomy

Eukaryota; Opisthokonta; Metazoa; Eumetazoa; Bilateria; Protostomia; Ecdysozoa; Panarthropoda; Arthropoda; Mandibulata; Pancrustacea; Hexapoda; Insecta; Dicondylia; Pterygota; Neoptera; Endopterygota; Hymenoptera; Apocrita; Ichneumonoidea; Braconidae; Rogadinae; *Aleiodes*; *Aleiodes testaceus* ( Telenga, 1941) (NCBI:txid422171).

## Background

*Aleiodes testaceus* is a koinobiont endoparasitoid braconid wasp, widely distributed across much of Europe, although less so in the North. Found in areas with trees and shrubby plants, until relatively recently it was rarely collected in the UK; however, the increasing use of light traps by lepidopterists has revealed that it is relatively common in southern England ( van Achterberg & Shaw, 2016). Over-wintering females can sometimes be beaten easily from coniferous trees in the winter.

A small, nearly uniformly brownish yellow (testaceous) rogadine braconid, *A. testaceus* is superficially similar to various other *Aleiodes* species. It can be recognised as a species of the subfamily Rogadinae by a combination of the cyclostome face, with a circular opening above the mandibles; three submarginal cells in the fore wing; and the spiracles of the second and third metasomal tergites within the notum and surrounded by sculpture, rather than lower on the epipleurae. Most European Rogadinae belong to the genus *Aleiodes* and within this very large genus, *A. testaceus* can be identified by its colour pattern (testaceous with a darker brown stripe laterally), granulate mesopleuron and rather short second submarginal cell. *Aleiodes testaceus* can be identified using van Achterberg and Shaw (2016). The name ‘ *testaceus*’ in the subfamily Rogadinae has often been ascribed to Spinola and used for a variety of testaceous species, but this has been the result of misidentifications. The relatively short second submarginal cell was probably the reason for Telenga (1941) describing this species in the genus *Heterogamus*, but van Achterberg and Shaw (2016) demonstrated that this is a species of *Aleiodes*.

As with other species of the genus *Aleiodes*, *A. testaceus* is known as a ‘mummy-wasp’ due to the hardened and partly shrunken structure (‘mummy’) that it causes its caterpillar hosts to form whilst it pupates. This process of ‘mummification’ is likely a form of protection for the developing wasp ( Zaldívar-Riverón *et al.*, 2008). *Aleiodes testaceus* is a parasitoid of pug-moths (Lepidoptera: Geometridae), with reliable host records reported by van Achterberg and Shaw (2016). Wild-collected specimens have been reared from *Eupithecia dodoneata*, *Chloroclystis v-ata* and *Gymnoscelis rufifasciata*. The species appears to be plurivoltine, with adults on the wing in the late spring and summer, mainly between June and September. Adults emerge from their hosts in the same year that mummification of the host occurs, but females over-winter as adults ( van Achterberg & Shaw, 2016). This genome will provide insights into host range evolution within parasitoid wasps and other traits regarding host use, enabling explicit hypotheses of host range evolution (e.g. Shaw, 2002) to be tested.

## Genome sequence report

**Figure 1.**
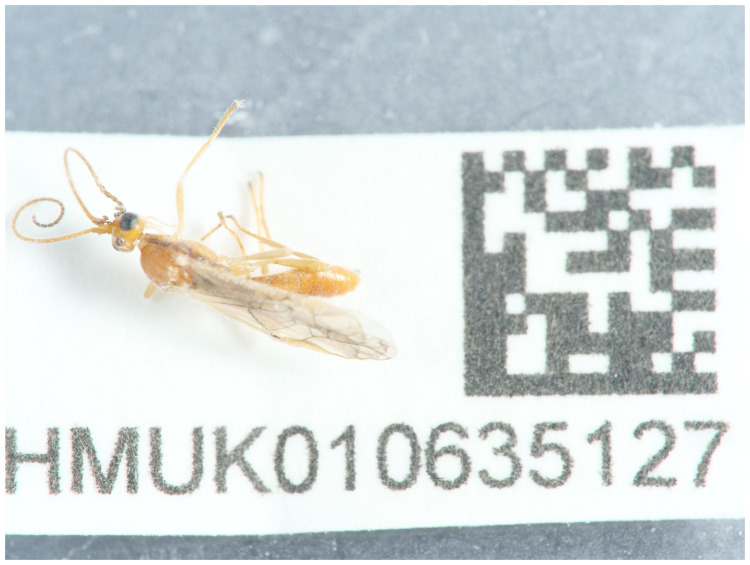
Photograph of the *Aleiodes testaceus* (iyAleTest2) specimen used for genome sequencing.

The genome of an adult female *Aleiodes testaceus* ( Figure 1) was sequenced using Pacific Biosciences single-molecule HiFi long reads, generating a total of 22.58 Gb (gigabases) from 1.65 million reads, providing approximately 199-fold coverage. Primary assembly contigs were scaffolded with chromosome conformation Hi-C data, which produced 116.98 Gbp from 774.68 million reads, yielding an approximate coverage of 1057-fold. Specimen and sequencing information is summarised in Table 1.

**Table 1.** Specimen and sequencing data for *Aleiodes testaceus*.

Project information			
**Study title**	Aleiodes testaceus		
**Umbrella BioProject**	PRJEB61692		
**Species**	*Aleiodes testaceus*		
**BioSample**	SAMEA111458708		
**NCBI taxonomy ID**	422171		
Specimen information			
**Technology**	**ToLID**	**BioSample accession**	**Organism part**
**PacBio long read sequencing**	iyAleTest2	SAMEA111458770	Whole organism
**Hi-C sequencing**	iyAleTest2	SAMEA111458770	Whole organism
**RNA sequencing**	iyAleTest1	SAMEA7696486	Whole organism
Sequencing information			
**Platform**	**Run accession**	**Read count**	**Base count (Gb)**
**Hi-C Illumina NovaSeq 6000**	ERR11439619	7.75e+08	116.98
**PacBio Sequel IIe**	ERR11279100	1.65e+06	22.58
**RNA Illumina NovaSeq 6000**	ERR12245569	6.71e+07	10.14

**Figure 2.**
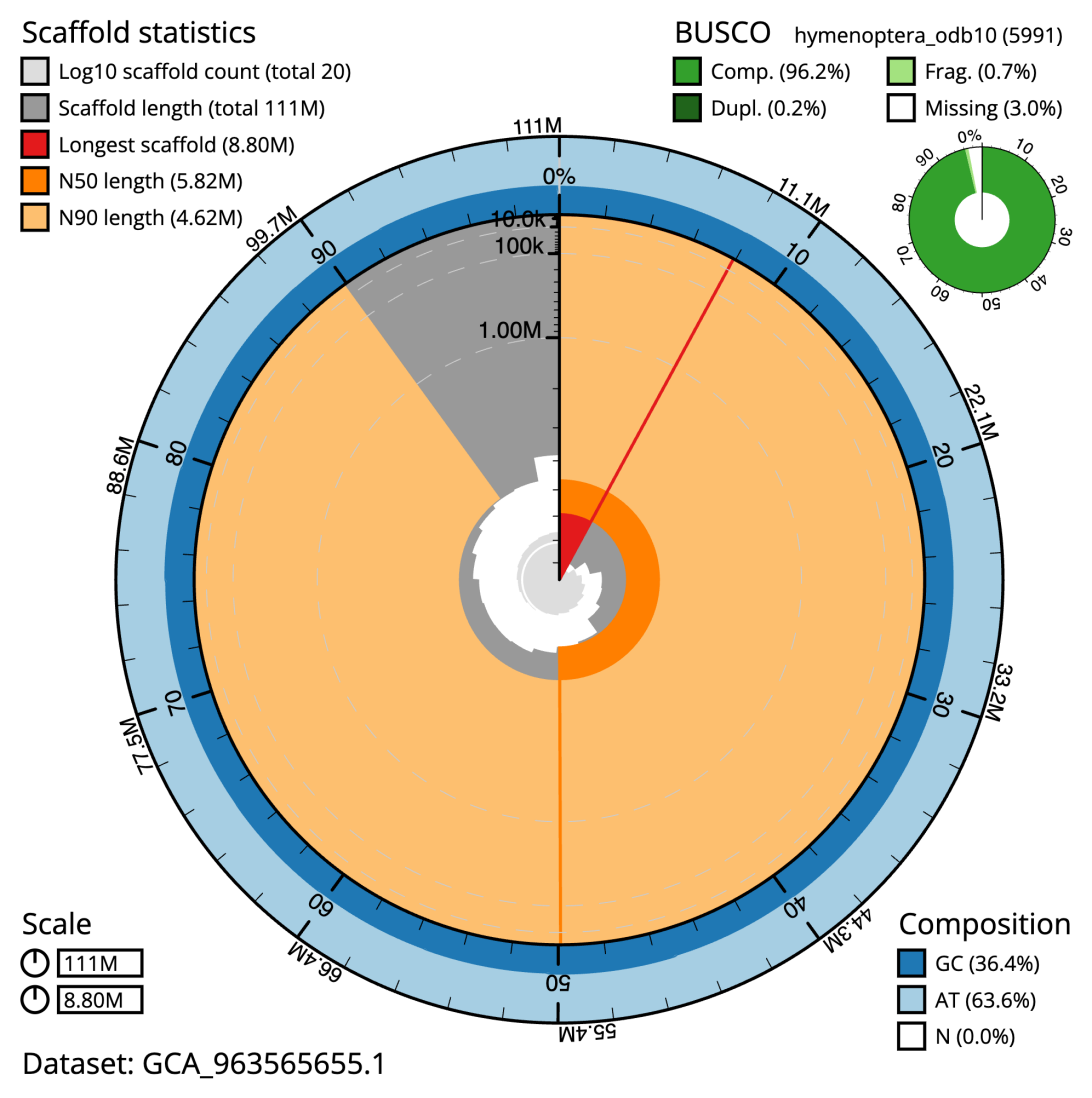
Genome assembly of *Aleiodes testaceus*, iyAleTest2.1: metrics. The BlobToolKit snail plot shows N50 metrics and BUSCO gene completeness. The main plot is divided into 1,000 size-ordered bins around the circumference with each bin representing 0.1% of the 110,733,156 bp assembly. The distribution of scaffold lengths is shown in dark grey with the plot radius scaled to the longest scaffold present in the assembly (8,797,196 bp, shown in red). Orange and pale-orange arcs show the N50 and N90 scaffold lengths (5,816,407 and 4,621,743 bp), respectively. The pale grey spiral shows the cumulative scaffold count on a log scale with white scale lines showing successive orders of magnitude. The blue and pale-blue area around the outside of the plot shows the distribution of GC, AT and N percentages in the same bins as the inner plot. A summary of complete, fragmented, duplicated and missing BUSCO genes in the hymenoptera_odb10 set is shown in the top right. An interactive version of this figure is available at https://blobtoolkit.genomehubs.org/view/Aleiodes_testaceus/dataset/GCA_963565655.1/snail.

**Figure 3.**
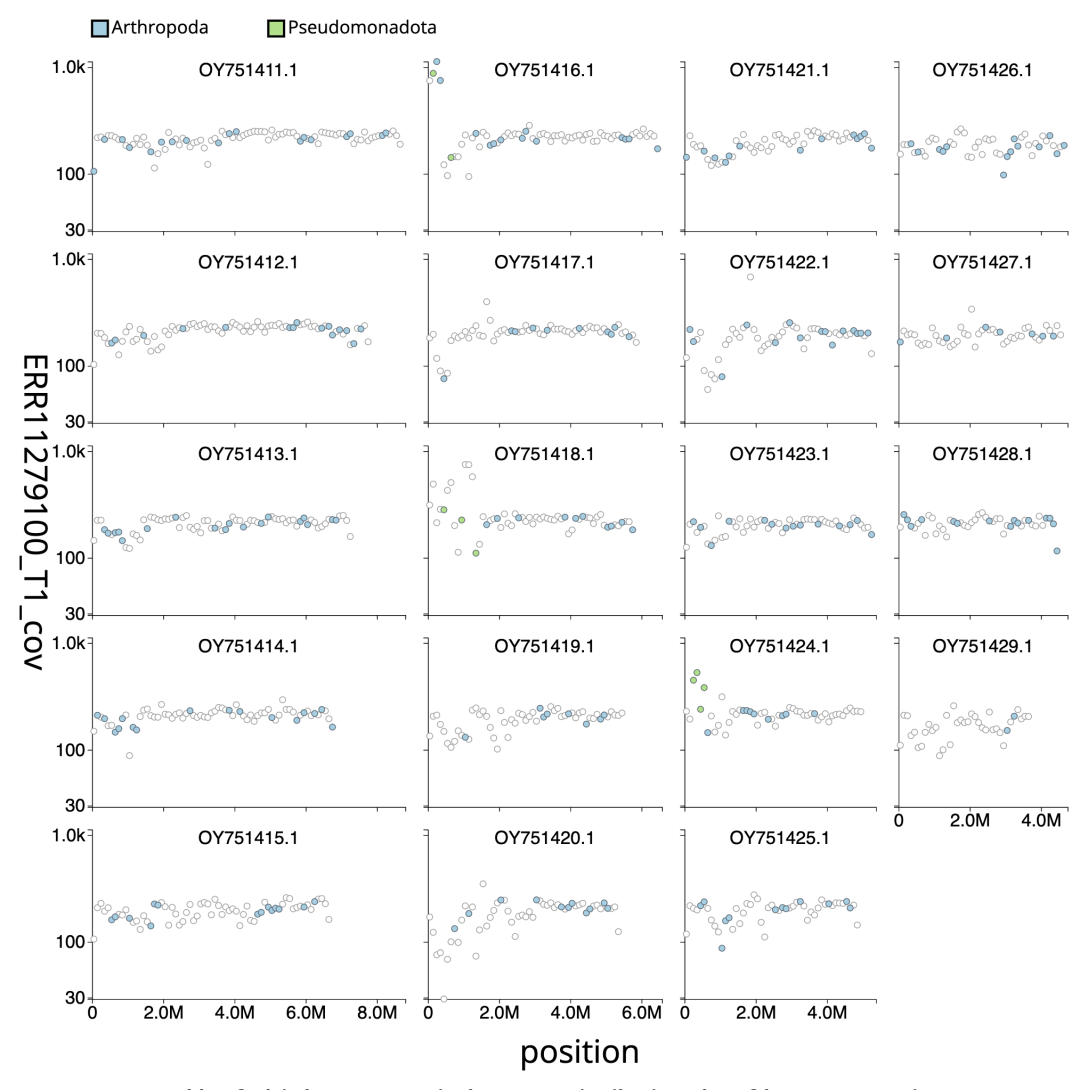
Genome assembly of *Aleiodes testaceus*, iyAleTest2.1: Distribution plot of base coverage in ERR11279100 against position for sequences in assembly GCA_963565655.1. Windows of 100kb are coloured by phylum. The assembly has been filtered to exclude sequences with length < 2,550,000. The interactive version can be viewed here.

**Figure 4.**
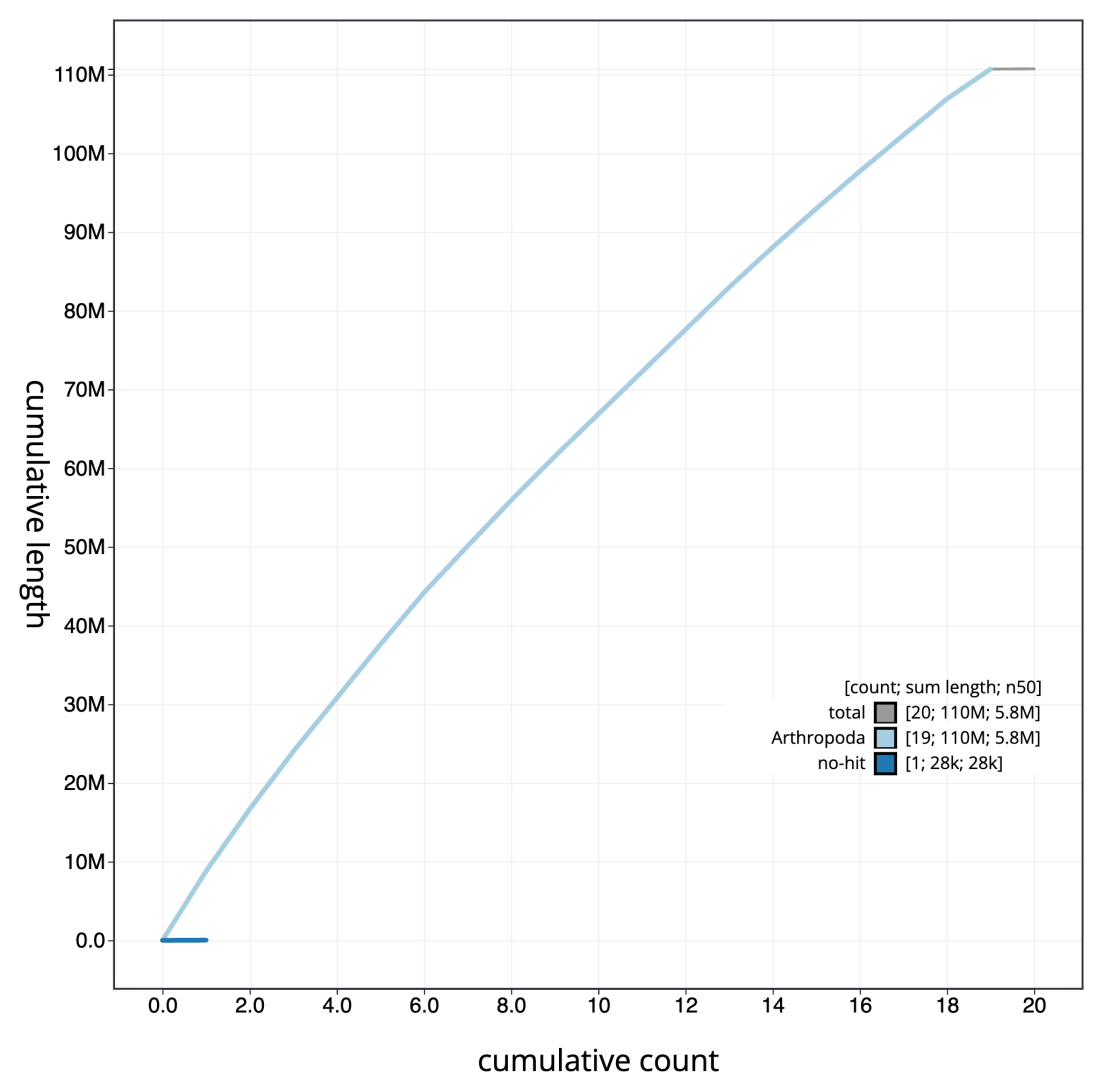
Genome assembly of *Aleiodes testaceus* iyAleTest2.1: BlobToolKit cumulative sequence plot. The grey line shows cumulative length for all sequences. Coloured lines show cumulative lengths of sequences assigned to each phylum using the buscogenes taxrule. An interactive version of this figure is available at https://blobtoolkit.genomehubs.org/view/Aleiodes_testaceus/dataset/GCA_963565655.1/cumulative.

**Figure 5.**
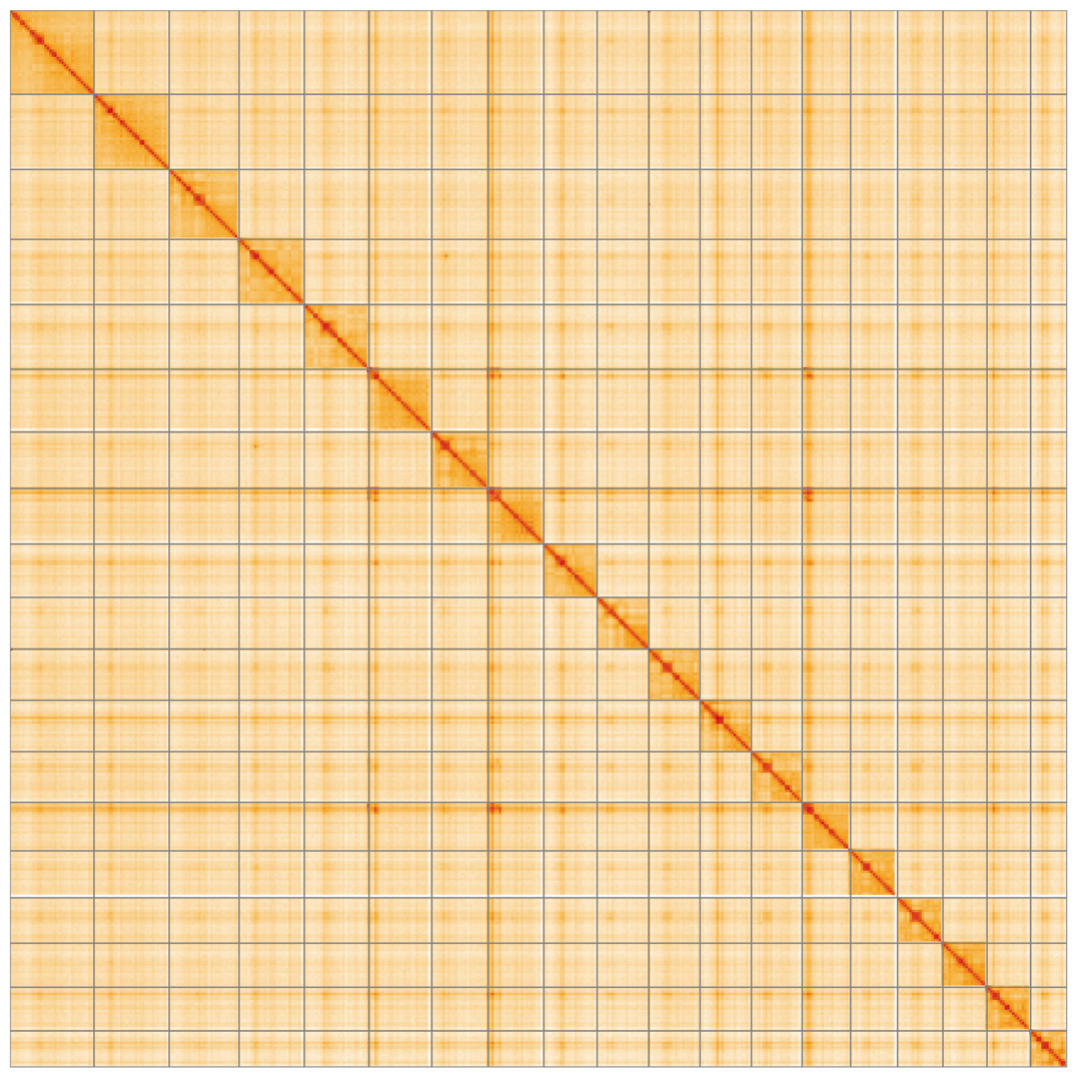
Genome assembly of *Aleiodes testaceus* iyAleTest2.1: Hi-C contact map of the iyAleTest2.1 assembly, visualised using HiGlass. Chromosomes are shown in order of size from left to right and top to bottom. An interactive version of this figure may be viewed at https://genome-note-higlass.tol.sanger.ac.uk/l/?d=djBJczfHSPqjNcIobRUNcw.

Manual assembly curation corrected 23 missing joins or mis-joins and 3 haplotypic duplications, reducing the scaffold number by 31.03%, and increasing the scaffold N50 by 4.14%. The final assembly has a total length of 110.70 Mb in 19 sequence scaffolds with a scaffold N50 of 5.8 Mb ( Table 2). The total count of gaps in the scaffolds is 49. The snail plot in Figure 2 provides a summary of the assembly statistics, while Figure 3 shows the distribution of assembly scaffolds based on base coverage across chromosomes. The cumulative assembly plot in Figure 4 shows curves for subsets of scaffolds assigned to different phyla. Most (99.97%) of the assembly sequence was assigned to 19 chromosomal-level scaffolds. Chromosome-scale scaffolds confirmed by the Hi-C data are named in order of size ( Figure 5; Table 3). While not fully phased, the assembly deposited is of one haplotype. Contigs corresponding to the second haplotype have also been deposited. The mitochondrial genome was also assembled and can be found as a contig within the multifasta file of the genome submission.

**Table 2.** Genome assembly data for *Aleiodes testaceus*, iyAleTest2.1.

Genome assembly		
Assembly name	iyAleTest2.1	
Assembly accession	GCA_963565655.1	
*Accession of alternate * *haplotype*	*GCA_963564045.1*	
Span (Mb)	110.70	
Number of contigs	69	
Contig N50 length (Mb)	3.0	
Number of scaffolds	19	
Scaffold N50 length (Mb)	5.8	
Longest scaffold (Mb)	8.8	
Assembly metrics *		*Benchmark*
Consensus quality (QV)	63.7	*≥ 50*
*k*-mer completeness	100.0%	*≥ 95%*
BUSCO **	C:95.9%[S:95.7%,D:0.2%], F:1.0%,M:3.1%,n:5,991	*C ≥ 95%*
Percentage of assembly mapped to chromosomes	99.97%	*≥ 95%*
Sex chromosomes	None	*localised * *homologous pairs*
Organelles	Mitochondrial genome: 28.0 kb	*complete single * *alleles*
Genome annotation of assembly GCA_963565655.1 at Ensembl		
Number of protein- coding genes	10,520	
Number of non-coding genes	1,001	
Number of gene transcripts	16,816	

* Assembly metric benchmarks are adapted from column VGP-2020 of “Table 1: Proposed standards and metrics for defining genome assembly quality” from Rhie *et al.* (2021). ** BUSCO scores based on the hymenoptera_odb10 BUSCO set using version 5.4.3. C = complete [S = single copy, D = duplicated], F = fragmented, M = missing, n = number of orthologues in comparison. A full set of BUSCO scores is available at https://blobtoolkit.genomehubs.org/view/Aleiodes_testaceus/dataset/GCA_963565655.1/busco.

**Table 3.** Chromosomal pseudomolecules in the genome assembly of *Aleiodes testaceus*, iyAleTest2.

INSDC accession	Name	Length (Mb)	GC%
OY751411.1	1	8.8	36.0
OY751412.1	2	7.87	36.5
OY751413.1	3	7.33	35.5
OY751414.1	4	6.83	36.0
OY751415.1	5	6.78	36.0
OY751416.1	6	6.58	36.5
OY751417.1	7	5.91	36.0
OY751418.1	8	5.82	37.5
OY751419.1	9	5.59	36.5
OY751420.1	10	5.41	36.0
OY751421.1	11	5.38	36.5
OY751422.1	12	5.36	36.0
OY751423.1	13	5.31	36.5
OY751424.1	14	5.09	37.5
OY751425.1	15	4.91	36.5
OY751426.1	16	4.75	36.5
OY751427.1	17	4.62	36.0
OY751428.1	18	4.58	37.0
OY751429.1	19	3.79	37.0
OY751430.1	MT	0.03	11.0

The estimated Quality Value (QV) of the final assembly is 63.7 with *k*-mer completeness of 100.0%, and the assembly has a BUSCO v5.4.3 completeness of 95.9% (single = 95.7%, duplicated = 0.2%), using the hymenoptera_odb10 reference set ( *n* = 5,991).

Metadata for specimens, BOLD barcode results, spectra estimates, sequencing runs, contaminants and pre-curation assembly statistics are given at https://links.tol.sanger.ac.uk/species/422171.

## Genome annotation report

The *Aleiodes testaceus* genome assembly (GCA_963565655.1) was annotated at the European Bioinformatics Institute (EBI) on Ensembl Rapid Release. The resulting annotation includes 16,816 transcribed mRNAs from 10,520 protein-coding and 1,001 non-coding genes ( Table 2; https://rapid.ensembl.org/Aleiodes_testaceus_GCA_963565655.1/Info/Index). The average transcript length is 6,647.33. There are 1.46 coding transcripts per gene and 5.56 exons per transcript.

## Methods

### Sample acquisition

Two adult female *Aleiodes testaceus* were collected from a garden in Tonbridge, England, UK (latitude 51.19, longitude 0.29) on 2020-08-20 and 2021-07-08 using actinic light. The specimens were collected and identified by Gavin Broad (Natural History Museum), and preserved by dry freezing at –80 °C. Specimen NHMUK014425700 (ToLID iyAleTest2) was used for genome sequencing and Hi-C scaffolding, and specimen NHMUK010635127 (ToLID iyAleTest1) was used for RNA sequencing.

In addition to identification based on morphology, the species taxonomy was verified by DNA barcoding soon after collection, according to the framework developed by Twyford *et al*. (2024). A small sample was dissected from the specimens and stored in ethanol. The tissue was lysed, and the COI marker region was amplified by PCR. Amplicons were sequenced and compared to the BOLD database, confirming the species identification ( Crowley *et al.*, 2023). The standard operating procedures for the Darwin Tree of Life barcoding have been deposited on protocols.io ( Beasley *et al.*, 2023). The remaining parts of the specimen were shipped on dry ice to the Wellcome Sanger Institute (WSI). A DNA barcode was also generated from the PacBio sequencing data at a later stage for sample tracking through the genome production pipeline at the WSI ( Twyford *et al.*, 2024).

### Nucleic acid extraction

The workflow for high molecular weight (HMW) DNA extraction at the Wellcome Sanger Institute (WSI) Tree of Life Core Laboratory includes a sequence of core procedures: sample preparation; sample homogenisation, DNA extraction, fragmentation, and clean-up. In sample preparation, the iyAleTest2 sample was weighed and dissected on dry ice ( Jay *et al.*, 2023). Tissue from the whole organism was homogenised using a PowerMasher II tissue disruptor ( Denton *et al.*, 2023a), setting aside tissue for Hi-C sequencing.

HMW DNA was extracted in the WSI Scientific Operations core using the Automated MagAttract v2 protocol ( Oatley *et al.*, 2023). The DNA was sheared into an average fragment size of 12–20 kb in a Megaruptor 3 system with speed setting 31 ( Bates *et al.*, 2023). Sheared DNA was purified by solid-phase reversible immobilisation ( Strickland *et al.*, 2023): in brief, the method employs a 1.8X ratio of AMPure PB beads to sample to eliminate shorter fragments and concentrate the DNA. The concentration of the sheared and purified DNA was assessed using a Nanodrop spectrophotometer and Qubit Fluorometer using the Qubit dsDNA High Sensitivity Assay kit. Fragment size distribution was evaluated by running the sample on the FemtoPulse system.

RNA was extracted from whole organism tissue of iyAleTest1 in the Tree of Life Laboratory at the WSI using the RNA Extraction: Automated MagMax™ *mir*Vana protocol ( do Amaral *et al.*, 2023). The RNA concentration was assessed using a Nanodrop spectrophotometer and a Qubit Fluorometer using the Qubit RNA Broad-Range Assay kit. Analysis of the integrity of the RNA was done using the Agilent RNA 6000 Pico Kit and Eukaryotic Total RNA assay.

Protocols developed by the WSI Tree of Life laboratory are publicly available on protocols.io ( Denton *et al.*, 2023b).

### Sequencing

Pacific Biosciences HiFi circular consensus DNA sequencing libraries were constructed according to the manufacturers’ instructions. DNA sequencing was performed by the Scientific Operations core at the WSI on a Pacific Biosciences Sequel IIe instrument. Hi-C data were also generated from remaining tissue of iyAleTest2 using the Arima-HiC v2 kit. The Hi-C sequencing was performed using paired-end sequencing with a read length of 150 bp on the Illumina NovaSeq 6000 instrument.

### Genome assembly, curation and evaluation

***Assembly***

Original assembly of HiFi reads is performed using Hifiasm ( Cheng *et al.*, 2021) with the --primary option. Haplotypic duplications were identified and removed with purge_dups ( Guan *et al.*, 2020). Hi-C reads are further mapped with bwa-mem2 ( Vasimuddin *et al.*, 2019) to the primary contigs, which are further scaffolded using the provided Hi-C data ( Rao *et al.*, 2014) in YaHS ( Zhou *et al.*, 2023) using the --break option. Scaffolded assemblies are evaluated using Gfastats ( Formenti *et al.*, 2022), BUSCO ( Manni *et al.*, 2021) and MERQURY.FK ( Rhie *et al.*, 2020).

The mitochondrial genome was assembled using MitoHiFi ( Uliano-Silva *et al.*, 2023), which runs MitoFinder ( Allio *et al.*, 2020) or MITOS ( Bernt *et al.*, 2013) and uses these annotations to select the final mitochondrial contig and to ensure the general quality of the sequence.

***Assembly curation***

The assembly was decontaminated using the Assembly Screen for Cobionts and Contaminants (ASCC) pipeline (article in preparation). Flat files and maps used in curation were generated in TreeVal ( Pointon *et al.*, 2023). Manual curation was primarily conducted using PretextView ( Harry, 2022), with additional insights provided by JBrowse2 ( Diesh *et al.*, 2023) and HiGlass ( Kerpedjiev *et al.*, 2018). Scaffolds were visually inspected and corrected as described by Howe *et al*. (2021). Any identified contamination, missed joins, and mis-joins were corrected, and duplicate sequences were tagged and removed. The process is documented at https://gitlab.com/wtsi-grit/rapid-curation (article in preparation).

***Evaluation of the final assembly***

The final assembly was post-processed and evaluated with the three Nextflow ( Di Tommaso *et al.*, 2017) DSL2 pipelines “sanger-tol/readmapping” ( Surana *et al.*, 2023a), “sanger-tol/genomenote” ( Surana *et al.*, 2023b), and “sanger-tol/blobtoolkit” ( Muffato *et al.*, 2024). The pipeline sanger-tol/readmapping aligns the Hi-C reads with bwa-mem2 ( Vasimuddin *et al.*, 2019) and combines the alignment files with SAMtools ( Danecek *et al.*, 2021). The sanger-tol/genomenote pipeline transforms the Hi-C alignments into a contact map with BEDTools ( Quinlan & Hall, 2010) and the Cooler tool suite ( Abdennur & Mirny, 2020), which is then visualised with HiGlass ( Kerpedjiev *et al.*, 2018). It also provides statistics about the assembly with the NCBI datasets ( Sayers *et al.*, 2024) report, computes *k*-mer completeness and QV consensus quality values with FastK and MERQURY.FK, and a completeness assessment with BUSCO ( Manni *et al.*, 2021).

The sanger-tol/blobtoolkit pipeline is a Nextflow port of the previous Snakemake Blobtoolkit pipeline ( Challis *et al.*, 2020). It aligns the PacBio reads with SAMtools and minimap2 ( Li, 2018) and generates coverage tracks for regions of fixed size. In parallel, it queries the GoaT database ( Challis *et al.*, 2023) to identify all matching BUSCO lineages to run BUSCO ( Manni *et al.*, 2021). For the three domain-level BUSCO lineage, the pipeline aligns the BUSCO genes to the Uniprot Reference Proteomes database ( Bateman *et al.*, 2023) with DIAMOND ( Buchfink *et al.*, 2021) blastp. The genome is also split into chunks according to the density of the BUSCO genes from the closest taxonomically lineage, and each chunk is aligned to the Uniprot Reference Proteomes database with DIAMOND blastx. Genome sequences that have no hit are then chunked with seqtk and aligned to the NT database with blastn ( Altschul *et al.*, 1990). All those outputs are combined with the blobtools suite into a blobdir for visualisation.

The evaluation pipelines were developed using the nf-core tooling ( Ewels *et al.*, 2020), use MultiQC ( Ewels *et al.*, 2016), and make extensive use of the Conda package manager, the Bioconda initiative ( Grüning *et al.*, 2018), the Biocontainers infrastructure ( da Veiga Leprevost *et al.*, 2017), and the Docker ( Merkel, 2014) and Singularity ( Kurtzer *et al.*, 2017) containerisation solutions.

Table 4 contains a list of relevant software tool versions and sources.

**Table 4.** Software tools: versions and sources.

Software tool	Version	Source
BEDTools	2.30.0	https://github.com/arq5x/bedtools2
BLAST	2.14.0	ftp://ftp.ncbi.nlm.nih.gov/blast/executables/blast+/
BlobToolKit	4.3.7	https://github.com/blobtoolkit/blobtoolkit
BUSCO	5.4.3 and 5.5.0	https://gitlab.com/ezlab/busco
bwa-mem2	2.2.1	https://github.com/bwa-mem2/bwa-mem2
Cooler	0.8.11	https://github.com/open2c/cooler
DIAMOND	2.1.8	https://github.com/bbuchfink/diamond
fasta_windows	0.2.4	https://github.com/tolkit/fasta_windows
FastK	427104ea91c78c3b8b8b49f1a7d6bbeaa869ba1c	https://github.com/thegenemyers/FASTK
Gfastats	1.3.6	https://github.com/vgl-hub/gfastats
GoaT CLI	0.2.5	https://github.com/genomehubs/goat-cli
Hifiasm	0.19.8-r603	https://github.com/chhylp123/hifiasm
HiGlass	44086069ee7d4d3f6f3f0012569789ec138f42b84aa4435 7826c0b6753eb28de	https://github.com/higlass/higlass
Merqury.FK	d00d98157618f4e8d1a9190026b19b471055b22e	https://github.com/thegenemyers/MERQURY.FK
MitoHiFi	3	https://github.com/marcelauliano/MitoHiFi
MultiQC	1.14, 1.17, and 1.18	https://github.com/MultiQC/MultiQC
NCBI Datasets	15.12.0	https://github.com/ncbi/datasets
Nextflow	23.04.0-5857	https://github.com/nextflow-io/nextflow
PretextView	0.2	https://github.com/sanger-tol/PretextView
purge_dups	1.2.5	https://github.com/dfguan/purge_dups
samtools	1.16.1, 1.17, and 1.18	https://github.com/samtools/samtools
sanger-tol/ ascc	-	https://github.com/sanger-tol/ascc
sanger-tol/genomenote	1.1.1	https://github.com/sanger-tol/genomenote
sanger-tol/ readmapping	1.2.1	https://github.com/sanger-tol/readmapping
Seqtk	1.3	https://github.com/lh3/seqtk
Singularity	3.9.0	https://github.com/sylabs/singularity
TreeVal	1.0.0	https://github.com/sanger-tol/treeval
YaHS	1.2a.2	https://github.com/c-zhou/yahs

### Genome annotation

The Ensembl Genebuild annotation system ( Aken *et al.*, 2016) was used to generate annotation for the *Aleiodes testaceus* assembly (GCA_963565655.1) in Ensembl Rapid Release at the EBI. Annotation was created primarily through alignment of transcriptomic data to the genome, with gap filling via protein-to-genome alignments of a select set of proteins from UniProt ( UniProt Consortium, 2019).

### Wellcome Sanger Institute – Legal and Governance

The materials that have contributed to this genome note have been supplied by a Darwin Tree of Life Partner. The submission of materials by a Darwin Tree of Life Partner is subject to the **‘Darwin Tree of Life Project Sampling Code of Practice’**, which can be found in full on the Darwin Tree of Life website here. By agreeing with and signing up to the Sampling Code of Practice, the Darwin Tree of Life Partner agrees they will meet the legal and ethical requirements and standards set out within this document in respect of all samples acquired for, and supplied to, the Darwin Tree of Life Project.

Further, the Wellcome Sanger Institute employs a process whereby due diligence is carried out proportionate to the nature of the materials themselves, and the circumstances under which they have been/are to be collected and provided for use. The purpose of this is to address and mitigate any potential legal and/or ethical implications of receipt and use of the materials as part of the research project, and to ensure that in doing so we align with best practice wherever possible. The overarching areas of consideration are:

•   Ethical review of provenance and sourcing of the material

•   Legality of collection, transfer and use (national and international)

Each transfer of samples is further undertaken according to a Research Collaboration Agreement or Material Transfer Agreement entered into by the Darwin Tree of Life Partner, Genome Research Limited (operating as the Wellcome Sanger Institute), and in some circumstances other Darwin Tree of Life collaborators.

## Data Availability

European Nucleotide Archive: *Aleiodes testaceus*. Accession number PRJEB61692; https://identifiers.org/ena.embl/PRJEB61692 ( Wellcome Sanger Institute, 2023). The genome sequence is released openly for reuse. The *Aleiodes testaceus* genome sequencing initiative is part of the Darwin Tree of Life (DToL) project. All raw sequence data and the assembly have been deposited in INSDC databases. Raw data and assembly accession identifiers are reported in Table 1 and Table 2.

## Author information

Members of the Natural History Museum Genome Acquisition Lab are listed here: https://doi.org/10.5281/zenodo.7139035.

Members of the Darwin Tree of Life Barcoding collective are listed here: https://doi.org/10.5281/zenodo.4893703.

Members of the Wellcome Sanger Institute Tree of Life Management, Samples and Laboratory team are listed here: https://doi.org/10.5281/zenodo.10066175.

Members of Wellcome Sanger Institute Scientific Operations: Sequencing Operations are listed here: https://doi.org/10.5281/zenodo.10043364.

Members of the Wellcome Sanger Institute Tree of Life Core Informatics team are listed here: https://doi.org/10.5281/zenodo.10066637.

Members of the Tree of Life Core Informatics collective are listed here: https://doi.org/10.5281/zenodo.5013541.

Members of the Darwin Tree of Life Consortium are listed here: https://doi.org/10.5281/zenodo.4783558.
