# Supplementary material for: The genome sequence of a braconid wasp, Aleiodes testaceus (Telenga, 1941)
Source: Wellcome Open Res. 2024 Jul 26;9:408. [Version 1] doi: 10.12688/wellcomeopenres.22738.1 (PMC11445644; doi:10.12688/wellcomeopenres.22738.1)
Supplement: Peer review history

# Reviewer report: Reviewer response for version 1

**DOI:** 10.21956/wellcomeopenres.25041.r96967

**Published:** 2024-10-01

**Authors:** Amrita Srivathsan

**Affiliations:** 1 Museum für Naturkunde Berlin, Berlin, Germany

The manuscript describes genome assembly of a braconid wasp. I think the genome will be a useful resource and I appreciate the efforts taken to make the various protocols and resources openly accessible. I think there are a couple of things that could be better elaborated:

 1. For barcoding, it is stated "A small sample was dissected from the specimens and stored in ethanol. The tissue was lysed, and the COI marker region was amplified by PCR. Amplicons were sequenced and compared to the BOLD database, confirming the species identification": What were the primers used, what was the sequencing technology? The citations refer to general SOPs but not what was specifically done here.

 2. Can the accession numbers for barcodes for these 2 specimens be shared in the manuscript, perhaps in Table 1

 3. Since specimens were collected independently, was there any genetic variation between the two, at least based on the barcode?

 4. For BUSCO, it would be good to add the mention of hymenoptera_odb10 in methods for assessment of completeness as this is mentioned in results.

 5. Figure 3, I find the green points of plot interesting. Particularly, there is an initial section of e.g. OY751424 that seems to better match to bacteria. Is this plot based on what is described in methods as "The genome is also split into chunks according to the density of the BUSCO genes from the closest taxonomically lineage, and each chunk is aligned to the Uniprot Reference Proteomes database with DIAMOND blastx. Genome sequences that have no hit are then chunked with seqtk and aligned to the NT database with blastn ( Altschul *et al.*, 1990). All those outputs are combined with the blobtools suite into a blobdir for visualisation." It would be good to establish what these steps were for in the description as it ends there. Were these regions manually examined to assess if these are really bacterial matches or issues due to reference database completion? Are these regions being detected in contamination removal pipeline?

Are sufficient details of methods and materials provided to allow replication by others?

Partly

Is the rationale for creating the dataset(s) clearly described?

Yes

Are the datasets clearly presented in a useable and accessible format?

Partly

Are the protocols appropriate and is the work technically sound?

Yes

Reviewer Expertise:

barcoding, species discovery, species interactions

I confirm that I have read this submission and believe that I have an appropriate level of expertise to confirm that it is of an acceptable scientific standard, however I have significant reservations, as outlined above.

# Reviewer report: Reviewer response for version 1

**DOI:** 10.21956/wellcomeopenres.25041.r96964

**Published:** 2024-09-20

**Authors:** Shannon Hedtke

**Affiliations:** 1 Department of Environment and Genetics, La Trobe University, Bundoora, Australia

This article by Broad & Cunningham-Eurich presents a new genome for a very cool wasp, *Aleiodes testaceus*. It will aid in comparative genomic work on the evolution of parasitoid behavior. I appreciate that there is ample description of the methods, including links to pipelines and to interactive results.

**Comments:**

 (1) The 2nd paragraph, which is the taxonomic summary, could be revised to enhance linearity and reduce repetitive language ("can be identified/recognized", for example).

 (2) Figure 3 is clearly imported directly from the blob toolkit used to produce it. The y-axis should be labeled using words (i.e., coverage) and not what I presume was the name of the coverage file. The legend needs to describe what each dot represents (which will clarify why the dots are coloured). Finally, it was very difficult to tell the difference between the arthropod and bacteria coloured dots; I strongly recommend editing these so that the colours can more readily be distinguished.

 Is there any scope for describing why there are what appear to be bacterial sequences in four contigs, most at high coverage and all at the start of contigs? I was surprised given the level of curation described that there was still potential contamination.

 (3) Figure 4 has *a lot* of empty space. The blue coloured line was also very hard to discriminate from the grey.

Are sufficient details of methods and materials provided to allow replication by others?

Yes

Is the rationale for creating the dataset(s) clearly described?

Yes

Are the datasets clearly presented in a useable and accessible format?

Yes

Are the protocols appropriate and is the work technically sound?

Yes

Reviewer Expertise:

I research population and phylogenomics of invertebrates, including nematodes and insects.

I confirm that I have read this submission and believe that I have an appropriate level of expertise to confirm that it is of an acceptable scientific standard.

# Reviewer report: Reviewer response for version 1

**DOI:** 10.21956/wellcomeopenres.25041.r96164

**Published:** 2024-09-20

**Authors:** Li-Bin Ma, Xuan Jing

**Affiliations:** 1 Shaanxi Normal University, Xian, China; 2 Shaanxi Normal University, Xi'an, China

The paper presented a report on an insect's genomic data, and the overall work was rough, with major problems in both the data and the writing.

**Significant issues:**

 1. The samples were from different periods over two years, but the article does not mention whether impurities were treated. It is recommended that details of sample screening and quality control be added to the methods section to explain how potential differences due to different sampling years are handled;

 2. The genome annotations in the full paper mainly focus on structural annotations and lack functional annotations. It is suggested to add detailed annotations of protein-coding genes, cDNAs, exons, introns, and intergenic regions, especially functional information of protein-coding genes;

 3. The completion of the section on mitochondrial genome assembly is insufficient; if assembled, it is recommended that annotations be added;

 4. In the section on BUSCO assessment, there is no mention of the specific databases compared, it is suggested that the type of databases used by BUSCO (e.g. arthropod databases) be clarified in the text.

**Minor issues:**

 1. In Figure 2, there is a lack of units and inconsistent use of units throughout the text (e.g., what does “M” mean in “5.82M”?); in the figure, BUSCO stands for different scales of 'green', which is difficult to recognize.

 2. There are also problems with the italicization of species names and punctuation (e.g., the application of colons and semicolons) between different taxonomic orders.

Are sufficient details of methods and materials provided to allow replication by others?

Partly

Is the rationale for creating the dataset(s) clearly described?

Yes

Are the datasets clearly presented in a useable and accessible format?

Partly

Are the protocols appropriate and is the work technically sound?

Partly

Reviewer Expertise:

Insect systematics; genome assembly, annotation and analysis

We confirm that we have read this submission and believe that we have an appropriate level of expertise to confirm that it is of an acceptable scientific standard, however we have significant reservations, as outlined above.
